# Spectacles: Their Uses and Abuses in Long and Short Sightedness; and the Pathological Conditions Resulting from Their Irrational Employment

**Published:** 1850-12

**Authors:** 


Art. XI.—*Spectacles:* their Uses and Abuses in long and short sightedness; and the Pathological conditions resulting from their irrational employment. By J. Sichel, M. D., of the Faculties of Berlin and Paris; Clinical Professor of Diseases of the Eye; Officer of the Legion of Honor, etc., etc., etc. Translated from the French, by permission of the author, by Henry W. Williams, M.D., Fellow of the Massachusetts Medical Society. Boston: Phillips, Sampson, & Company. 1850.

We have, in the English language, many books about Spectacles, and scarcely one on the subject. With the exception of the little treatise, published by Mr. Cooper in 1847, we do not know of an English work on the subject that a physician can consult with any profit to himself, or commend to an inquirer with any degree of confidence. This is an astonishing state of things, for the information is almost in daily requisition. The defects that demand a remedy or aid for impaired vision, are so numerous, and vary so widely, that accurate and full knowledge of the causes of defective sight, and the exact means for relief are the essential parts of a physician's education. If his power to render proper aid were more frequently recognised than it is, and there were any good reason why people should have confidence in his assistance, many eyes that are screened with glasses, would rejoice in powers of their own, and many that are deficient with glasses, would feel their strength renewed with a proper adaptation of the crutches of the sight. If a man with an injury in his legs uses crutches, too long or too short, he must not only use them greatly to his personal discomfort, but to his injury.

Those who have studied the philosophy of light, and of the apparatus of vision, know the delicate character of the laws that operate in these matters. Every luminous body sends forth rays of light from a great multitude of points, and they spread out so much, that if it were not for that law of light, which compels rays to bend in passing through a medium of greater density, than that in which they spread, we should have perpetual difficulty. It is the province of the organs of sight to gather these rays together at a proper point, and deliver them in a proper condition to the retina, and where there is any failure in those functions, there is defective vision. To aid these failing powers, spectacles are usually resorted to, and often without any well defined knowledge of the nature of the cause that requires their assistance. Surely it is a matter of some moment to determine whether the defective vision is merely functional, or, as Mr. Abernethy would have said, from the stomach, or from an altered condition of the structure of the parts concerned in vision. If it is correctly determined that the cause of defective sight is one that needs the aid of glasses, it is an affair of great importance to decide upon the proper kind, adapted to the special case. Many eyes are incurably injured by the very means resorted to for their preservation.

The work before us is from the hand of a master of the subject, and is well worthy the attention of physicians. M. Sichel says: "the publication of these Clinical Lessons on Spectacles, and the pathological conditions consecutive to their irrational use, was commenced in 1845, in the *Annales d'Oculistique*, at Brussels. The present work contains many new and very important considerations, and a description of several frequent and grave maladies, of which no one had previously spoken; such as *presbytic amblyopia*, *congenital presbytic amblyopia*, *amblyopia produced by the use of too powerful glasses*, *muscæ volitantis caused by the abuse of spectacles.* These affections may be prevented, arrested in their development, or completely cured, by the use of properly selected spectacles. For these reasons I may recommend the perusal of these pages, not only to physicians, but also to opticians. The latter have it in their power, yet more than the former, to diminish the number of these maladies by judicious advice, or to augment it by the unreasonable concession of too powerful glasses. Unfortunately, they very generally continue the habit of commencing by too powerful numbers in the selection of spectacles, although this practice is contrary to their own interests."

We do not know that we can better express the character of M. Sichel's work, than by quoting the translator's preface. He says: "I have great satisfaction in availing myself of the kind permission of its distinguished author, [M. Sichel] and publishing in this country a translation of a work emanating from the highest authority in this department of medical science."

After expressing views similar to those we have written, on the general ignorance that prevails on the subjects treated of in the book, Dr. Williams says: "it is therefore, with a full conviction that this monograph will be generally welcomed, and will prove a guide of the highest utility, that its valuable pages, the results of mature observation, cultivated intellect, and wide experience, are offered to the profession in America."

We have rarely read any work that gave us a higher degree of pleasure than we derived from this. No one can read it without feeling that he is in the hands of a master of the subject, and few will rise from its perusul unimpressed with the great value of the instruction given by M. Sichel. It is a matter of astonishment that, in such an important matter as the use of spectacles for aiding vision, there are so few who attend to the essential steps in the great change about to be made. The first question should be, are spectacles necessary? If this is decided in the affirmative, the next important inquiry, and for serious investigation too, is, what kind are best adapted to the case? What kind will give present help without augmenting the defect, for which aid is sought. The volume before us gives ample information on these points, and upon a multitude of kindred topics, and we commend the work to the thoughtful study of the reader of these remarks.

The work of M. Sichel has been clothed in an excellent English garb by Dr. Williams, and we cordially thank him for this service to the profession. The publishers have disharged their duties very creditably.

We are indebted to the courtesy of Messrs. Beckwith & Morton for these various works, and copies of them may be obtained at their bookstore.

